# TRPM4 and TRPM5 Channels Share Crucial Amino Acid Residues for Ca^2+^ Sensitivity but Not Significance of PI(4,5)P_2_

**DOI:** 10.3390/ijms20082012

**Published:** 2019-04-24

**Authors:** Soichiro Yamaguchi, Akira Tanimoto, Shinsuke Iwasa, Ken-ichi Otsuguro

**Affiliations:** 1 Laboratory of Physiology, Department of Basic Veterinary Sciences, Faculty of Veterinary Medicine, Hokkaido University, Sapporo 060-0818, Japan; 2 Laboratory of Pharmacology, Department of Basic Veterinary Sciences, Faculty of Veterinary Medicine, Hokkaido University, Sapporo 060-0818, Japan; 1990.a.tanimoto@gmail.com (A.T.); ekusnihs1180@gmail.com (S.I.); otsuguro@vetmed.hokudai.ac.jp (K.O.)

**Keywords:** TRPM4, TRPM5, Ca^2+^-binding site, PI(4,5)P_2_, patch clamp, site-directed mutagenesis

## Abstract

Transient receptor potential melastatin member 4 (TRPM4) and 5 (TRPM5) channels are Ca^2+^-activated nonselective cation channels. Intracellular Ca^2+^ is the most important regulator for them to open, though PI(4,5)P_2_, a membrane phosphoinositide, has been reported to regulate their Ca^2+^-sensitivities. We previously reported that negatively-charged amino acid residues near and in the TRP domain are necessary for the normal Ca^2+^ sensitivity of TRPM4. More recently, a cryo-electron microscopy structure of Ca^2+^-bound (but closed) TRPM4 was reported, proposing a Ca^2+^-binding site within an intracellular cavity formed by S2 and S3. Here, we examined the functional effects of mutations of the amino acid residues related to the proposed Ca^2+^-binding site on TRPM4 and also TRPM5 using mutagenesis and patch clamp techniques. The mutations of the amino acid residues of TRPM4 and TRPM5 reduced their Ca^2+^-sensitivities in a similar way. On the other hand, intracellular applications of PI(4,5)P_2_ recovered Ca^2+^-sensitivity of desensitized TRPM4, but its effect on TRPM5 was negligible. From these results, the Ca^2+^-binding sites of TRPM4 and TRPM5 were shown to be formed by the same amino acid residues by functional analyses, but the impact of PI(4,5)P_2_ on the regulation of TRPM5 seemed to be smaller than that on the regulation of TRPM4.

## 1. Introduction

Transient receptor potential melastatin member 4 (TRPM4) and member 5 (TRPM5) channels are Ca^2+^-activated nonselective monovalent cation channels. They share 40% homology in their amino-acid sequences, so that they are the closest homologs among eight members of the TRPM family [1]. TRPM4 is expressed in a broad range of cells such as cardiac myocytes, immune cells, etc. [1]. Conversely, TRPM5 is expressed in a relatively small number of tissues with the highest expression in type II taste cells, which detect bitter, sweet and umami stimuli [1]. The activities of TRPM4 and TRPM5 influence the functions of the cells, where they are expressed, by depolarizing the membrane potential when they open by a rise in intracellular Ca^2+^ (Ca^2+^_i_) concentration ([Ca^2+^]_i_). The Ca^2+^-sensitivity of TRPM4 has been reported to be maintained by interaction with PI(4,5)P_2_, a major phosphoinositide in plasma membrane [2], and a depletion of PI(4,5)P_2_ caused desensitization of TRPM4 [3,4]. PI(4,5)P_2_ was also reported to have partially restored TRPM5 channel activity after its desensitization [5]. However, their opening is completely dependent on Ca^2+^_i_ because without Ca^2+^ they are unable to open even if there is a sufficient amount of PI(4,5)P_2_ [4,6].

**Figure 1:**
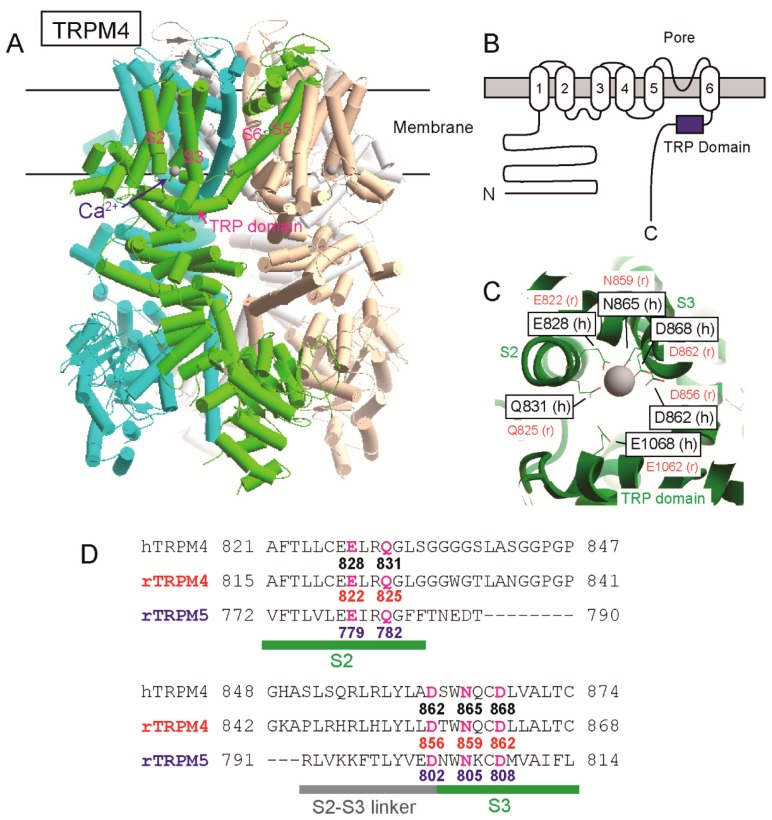
The Ca^2+^-binding site of transient receptor potential melastatin member 4 (TRPM4) proposed by a cryo-electron microscopy (cryo-EM) structure analysis. (**A**) A cryo-EM structure of Ca^2+^-bound human TRPM4 (hTRPM4) after [11] TRPM4 forms a tetramer. Each monomer is shown in different colors. (**B**) A membrane topology of TRPM4. (**C**) An enlarged bottom view of the Ca^2+^-binding site. Black letters and red letters indicate the amino acid residues of hTRPM4 and rat TRPM4 (rTRPM4), respectively. The gray ball is Ca^2+^. (**D**) An alignment of the amino acid sequences of hTRPM4 (GenBank #AAI32728.1), rTPRM4 (NP_001129701.1) and rat TRPM5 (rTRPM5) (NP_001178825.1) around the Ca^2+^-binding site. Magenta letters indicate the amino acids which were mutated in this study.

The position of a Ca^2+^-binding site of TRPM4 had been unknown for a long time since its cloning. However, as a result of site-directed mutagenesises and patch clamp analyses of rat TRPM4 (rTRPM4), we previously found that negatively charged amino acid residues near and in the TRP domain of the intracellular C-terminal tail are necessary for the normal Ca^2+^-sensitivity of rTRPM4 [6]. They are Asp^1049^ and Glu^1062^ of rTRPM4 and are conserved in the other Ca^2+^-sensitive TRPM channels (TRPM5, TRPM2 and TRPM8) [6,7,8]. More recently, several cryo-electron microscopy (cryo-EM) structures of TRPM4 were reported [9,10,11,12]. One of them is a Ca^2+^-bound structure of human TRPM4 (hTRPM4, [11]). Ca^2+^ was surrounded by four amino acid residues of the transmembrane segment 2 (S2) and 3 (S3) (Glu^828^, Gln^831^, Asn^865^, and Asp^868^ of hTRPM4) within a cavity accessible from cytosol (Figure 1, [11]). The glutamate in the TRP domain, which we reported, was located at the entrance of the cavity and assumed to enhance access of Ca^2+^ to the Ca^2+^-binding site through its negative charge (Figure 1, [9,11]). The cryo-EM structures of TRPM2 [13,14] and TRPM8 [15] supported that the four amino acid residues in S2 and S3 form their Ca^2+^-binding sites.

Although the proposed Ca^2+^-binding site is most likely to be necessary for TRPM4 to open, the possibility has not been fully proven. That is firstly because the Ca^2+^-bound structure of hTRPM4 was in a closed state although Ca^2+^ bound to the site [11]. Moreover, that is also because the importance of the four amino acid residues for Ca^2+^-sensitivity of TRPM4 has not been evaluated by functional analyses. Concerning TRPM5, although the amino acid residues forming the Ca^2+^-binding site of TRPM4 are conserved in TRPM5, the position of a Ca^2+^-binding site of TRPM5 has not been experimentally revealed. Therefore, a primary aim of this study is to reveal amino acid residues which form Ca^2+^-binding sites of TRPM4 and TRPM5 by functional analyses using mutagenesises, an inside-out patch clamp technique and a whole-cell patch clamp technique.

As a secondary aim of this study, we re-evaluated the effects of PI(4,5)P_2_ on rTRPM4 and rTRPM5. The PI(4,5)P_2_-binding site of TRPM4 was suggested to be located at the pre-S1 (i.e., just before S1) region in the intracellular N-terminal tail [16]. However, the similarity of the pre-S1 region between TRPM channels is low and the important amino acid residues for the binding of PI(4,5)P_2_ to TRPM4 are not conserved in the pre-S1 region of TPRM5 [15,16]. Therefore, the pre-S1 region of TRPM5 may not be able to function as a PI(4,5)P_2_ binding site. Additionally, there seems to be a difference between the extent of the effect of PI(4,5)P_2_ on TRPM4 and TRPM5 when they are compared in literature. As written in the initial report on the effect of PI(4,5)P_2_ on TRPM5, PI(4,5)P_2_ “partially” restored the sensitivity of the channel to Ca^2+^ [5]. On the other hand, PI(4,5)P_2_ completely restores Ca^2+^-sensitivity and channel activity of TRPM4 [3,4,6]. Therefore, in this study, we compared the effects of PI(4,5)P_2_ on rTRPM4 and rTRPM5 under the same condition using the same cell line, the same expression system, and the same inside-out patch clamp technique.

## 2. Results

### 2.1. Functional Analyses for the Amino Acid Residues Which Were Proposed to Form the Ca^2+^-Binding Site of TRPM4

#### 2.1.1. Mutations of the Amino Acid Residues Which Were Proposed to form the Ca^2+^-Binding Site Reduced the Ca^2+^-Sensitivity of rTRPM4

**Figure 2:**
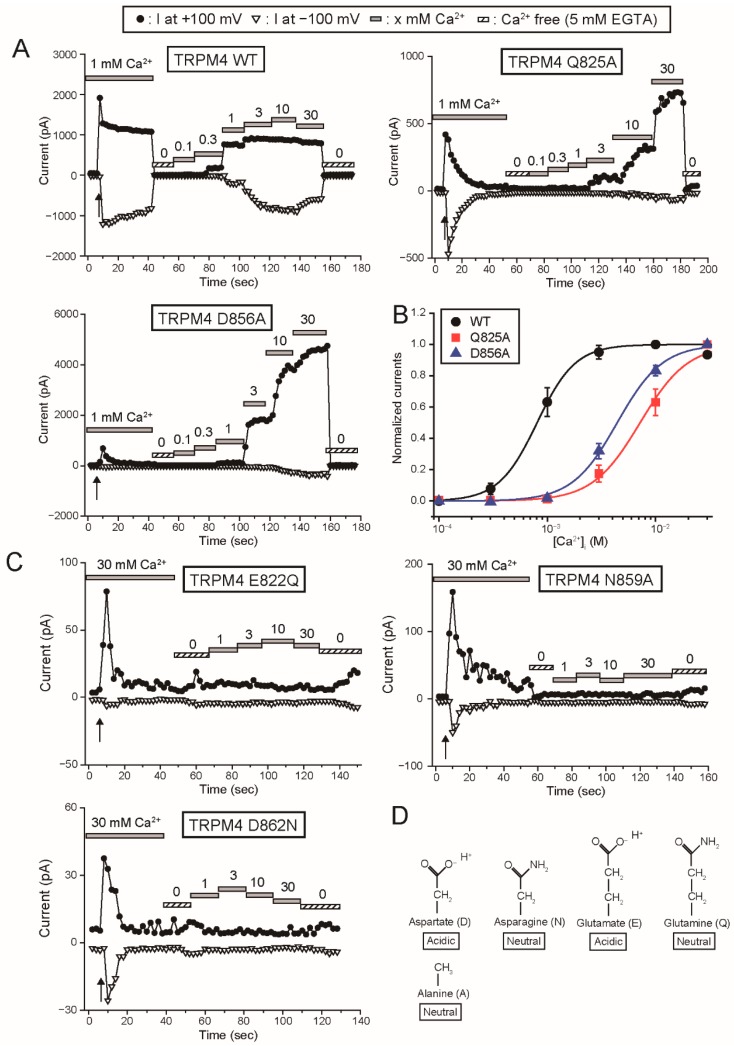
Mutations of the amino acid residues of the proposed Ca^2+^-binding site of rTRPM4 reduced the Ca^2+^-sensitivity. (**A**) Typical time courses of the changes in the currents at +100 mV (filled circles) and −100 mV (open inverted triangles) of wild-type (WT) rTRPM4, Q825A and D856A. After the current amplitudes became almost steady in the presence of 1 mM intracellular Ca^2+^ (Ca^2+^_i_), Ca^2+^_i_ concentrations ([Ca^2+^]_i_) were varied from 0 mM (chelated with 5 mM EGTA) to 30 mM. Arrows indicate the time of the patch excision. (**B**) Concentration-response curves (CRCs) for the effect of Ca^2+^ on WT TRPM4 (black circles), Q825A (red squares, and D856A (blue triangles). Current amplitudes at +100 mV were used for analyses. The shifts of CRCs by the mutations seemed to be underestimated, because the CRCs were drawn on the assumption that the current amplitudes at 30 mM Ca^2+^_i_ were maximal but their current amplitudes at 30 mM Ca^2+^_i_ were actually not saturated. (*n* = 7 or 8, each). (**C**) Typical time courses of the currents at +100 mV (filled circles) and −100 mV (open inverted triangles) of E822Q, N859A and D862N. Patch membranes were excised in the presence of 30 mM Ca^2+^_i_. Similar results were obtained repeatedly (*n* = 6 each). (**D**) Chemical structures of side chains of the amino acid residues which were mutated in this study.

**Figure 3:**
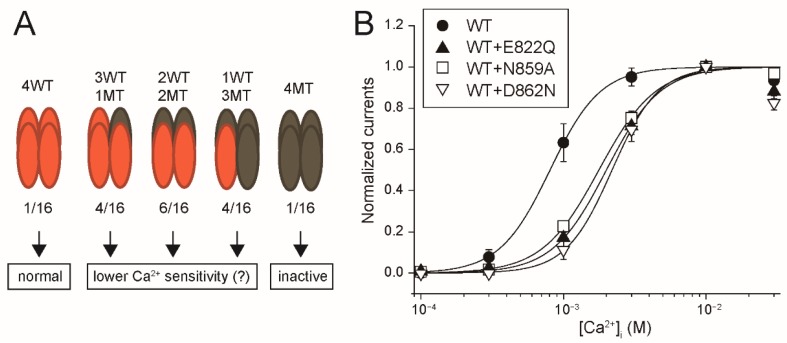
Co-expression of E822Q, N859A or D862N reduced Ca^2+^-sensitivity of WT rTRPM4. (**A**) A scheme for our working hypothesis. Orange ellipses are WT rTRPM4 monomers. Dark gray ellipses are mutant monomers (MT). The numbers under the ellipses show each occurrence frequency of the homomers and the heteromers when they are co-expressed evenly. (**B**) CRCs for the effect of Ca^2+^ on the currents at +100 mV, which were mediated by co-expressed WT and E822Q (filled triangles), WT and N859A (open squares) and WT and D862N (open inverted triangles) (*n* = 6 each). The data for WT (filled circles) is the same in Figure 2B. Typical time courses of the changes in their currents are shown in Supplementary Figure S1.

**Figure 4:**
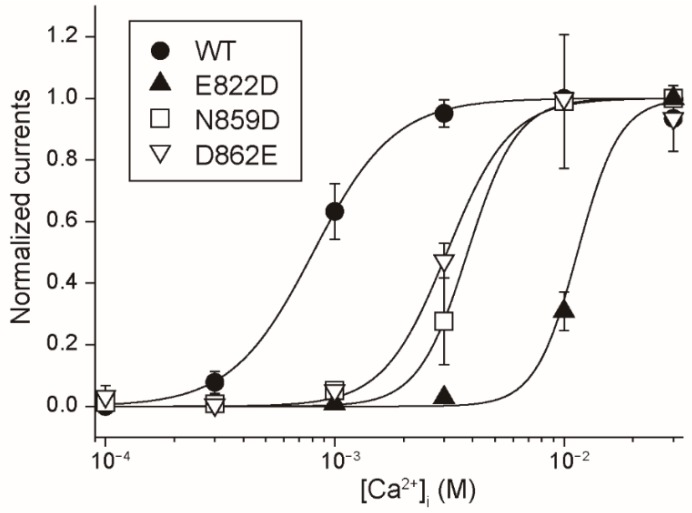
Mutations of Glu^822^, Asn^859^ and Asp^862^ to the negatively-charged amino acid residues reduced the Ca^2+^-sensitivity of rTRPM4. CRCs for the effect of Ca^2+^ on the currents at +100 mV, which were mediated by E822D (filled triangles), N859D (open squares) and D862E (open inverted triangles) (*n* = 8, 5 and 8, respectively) are shown. The data for WT (filled circles) is the same in Figure 2B. Typical time courses of the changes in their currents are shown in Supplementary Figure S2.

Firstly, we examined the effects of mutations of the amino acid residues at the shallow position of the proposed Ca^2+^-binding site on the Ca^2+^-sensitivity of rTRPM4. Gln^825^ of rTRPM4 seems to contact with Ca^2+^ based on the structure of Ca^2+^-bound hTRPM4 ([11], Figure 1C). Asp^865^ of rTRPM4 appears not to contact with Ca^2+^ but its negatively-charged side chain seems to face the pathway for Ca^2+^. We measured currents of rTRPM4, which was heterologously expressed in HEK293T cells, using an inside-out patch clamp technique similar to the previous report [6]. The rTRPM4 currents showed a rapid rundown after the patch excision because of desensitization, which was probably caused by the depletion of PI(4,5)P_2_ in the patch membrane (Figure 2A, [3,4,6]). The Ca^2+^-sensitivities of wild-type (WT) and mutant rTRPM4 were evaluated using the currents after the desensitization in order to reveal their affinities to Ca^2+^ while minimizing the influence of the changes in their affinities to PI(4,5)P_2_, if any. [Ca^2+^]_i_ was varied by changing the Ca^2+^ concentrations of bath solutions. Although the WT rTRPM4 current amplitudes were saturated at 3 mM Ca^2+^_i_, Q825A (Gln^825^ was mutated to alanine, Figure 2D) mutant and D856A (Asp^856^ → Ala) mutant required more than 3 mM Ca^2+^_i_ to show their currents (Figure 2A). Unlike WT TRPM4, the initial peak current amplitudes of Q825A and D856A were smaller than the current amplitudes in the presence of 30 mM Ca^2+^ (Figure 2A). That suggests that 1 mM Ca^2+^ was insufficient to evoke substantial currents of Q825A and D856A even at the moment of patch excisions due to their lower Ca^2+^-sensitivities. The concentration-response curves (CRCs) of Q825A and D856A were rightward shifted in comparison with that of WT (Figure 2B). Half maximal effective concentrations (EC_50_) for Ca^2+^ of WT rTRPM4, Q825A and D856A currents were 0.81, 7.19 and 4.42 mM, respectively.

Next, we examined the effects of mutations of the amino acid residues at the deep position of the proposed Ca^2+^-binding site. These amino acid residues (Glu^822^, Asn^859^ and Asp^862^ of rTRPM4) seem to contact with Ca^2+^ (Figure 1C). Glu^822^ and Asp^862^ of rTRPM4 were mutated to glutamine (E822Q) and asparagine (D862N), respectively, in order to eliminate the negative charge in their side chains (Figure 2D). Asn^859^ was mutated to alanine (N859A). At first, we tried to measure the mutant currents under the presence of 1 mM Ca^2+^_i_, which was able to evoke substantial WT rTRPM4 currents (Figure 2A). However, no detectable TPRM4 currents were measured from the cells which expressed these mutants (data not shown). Even in the presence of 30 mM Ca^2+^_i_, the transient currents were evoked only immediately after the patch excisions, and afterward no Ca^2+^_i_-dependent currents were measured (Figure 2C). From the cells which were transfected with an empty vector, such transient currents were not observed even in the presence of 30 mM Ca^2+^_i_ (data not shown). These results suggest that all of the mutations of the amino acid residues at the deep position of the proposed Ca^2+^-binding site reduced Ca^2+^-affinities of the mutants so much that the Ca^2+^-sensitivities of mutants were almost lost, especially after the desensitization.

#### 2.1.2. The Ca^2+^-Sensitivity of WT rTRPM4 was Reduced by Co-Expression of the Mutants in Which the Amino Acid Residues at the Deep Position of the Proposed Ca^2+^-Binding Site Were Mutated

The Ca^2+^-sensitivities of E822Q, N859A or D862N were unable to be precisely estimated because there were no currents after their desensitization (Figure 2C). Therefore, as another approach in examining whether these mutations reduced the Ca^2+^-affinity of rTRPM4, we evaluated the effects of co-expression of E822Q, N859A or D862N on the Ca^2+^-sensitivity of WT rTRPM4. Firstly, we explain our working hypothesis. When the tetramers of rTRPM4 are formed only by these mutants, the channels were almost inactive as already shown in Figure 2C. If the Ca^2+^-affinities of these mutants are lower than that of WT rTRPM4, the heteromer channels which are formed by WT and mutant TRPM4 will show the Ca^2+^-sensitivities lower than that of WT rTRPM4 homomers (Figure 3A, middle). Stochastically, the majority of the tetramers will be the heteromers (14/16, 87.5%). When WT rTRPM4 and these mutants are co-expressed, the currents are expected to be the mixture of WT rTRPM4 homomer currents and heteromer currents. Therefore, the co-expressed currents may exhibit rightward-shifted CRCs in comparison with that of solely expressed WT rTRPM4. As shown Figure 3B, the co-expression of E822Q, N859A or D862N shifted the CRCs for the effect of Ca^2+^ on WT rTRPM4. EC_50_ for Ca^2+^ of the co-expressed WT and E822Q currents, the co-expressed WT and N859A currents, and the co-expressed WT and D862N currents were 2.00, 1.78 and 2.19 mM, respectively. These results also suggest that the mutations of amino acid residues at the deep position of the proposed Ca^2+^-binding site reduced the Ca^2+^-affinity of rTRPM4.

#### 2.1.3. Mutations of the Amino Acid Residues at the Deep Position to Even the Negatively-Charged Amino Acid Residues Reduced the Ca^2+^-Sensitivity of rTRPM4

In order to further confirm that Glu^822^, Asn^859^ and Asp^862^ participate in forming the Ca^2+^-binding site of rTRPM4, we examined the influence of the mutations of these amino acid residues to negatively-charged amino acid residues. Glu^822^ and Asp^862^ were mutated to aspartate (E822D and N862D). Asp^862^ was mutated to glutamate (D862E). Although the negative charge of the residues remained in E822D and D862E or was added in N862D, the Ca^2+^-sensitivities of the mutants were lower than that of WT rTRPM4 (Figure 4). EC_50_ for Ca^2+^ of E822D, N859D and D862E currents were 11.5, 3.80 and 3.09 mM, respectively. Although there had been also a possibility that these mutations would rather increase the Ca^2+^-sensitivity, actually their Ca^2+^-sensitivities were reduced by the mutations. These results also indicate that Glu^822^, Asn^859^ and Asp^862^ participate in forming the Ca^2+^-binding site of rTRPM4. Additionally, these results suggest that the combination of these amino acid residues in WT rTRPM4 is the best for forming a high-affinity Ca^2+^-binding site, and the changes in the length of these side chains and in the number of negative charges impaired the Ca^2+^-affinity of the Ca^2+^-binding site.

### 2.2. The Normal Ca^2+^-Sensitivity of rTRPM5 Required the Same Amino Acid Residues with rTRPM4

#### 2.2.1. The Ca^2+^-Sensitivity of rTRPM5 was Reduced by the Mutations of the Negatively-Charged Amino Acid Residues near and in the TRP Domain, Which Were Previously Reported to be Necessary for the Normal Ca^2+^-Sensitivity of rTRPM4

Firstly, we cloned rat Trpm5 cDNA from the tongue epithelia. Although this is outside of the aims of this study, we found additional possible initiation codons (ATG) in the 5’ untranslated region (UTR) (Supplementary Figure S3A). However, from the results of patch clamp analyses and Western blot analyses, it was shown that the translation of rTRPM5 does not start, at least mainly, from these possible initiation codons in 5’ UTR (Supplementary Figures S3B–F).

**Figure 5:**
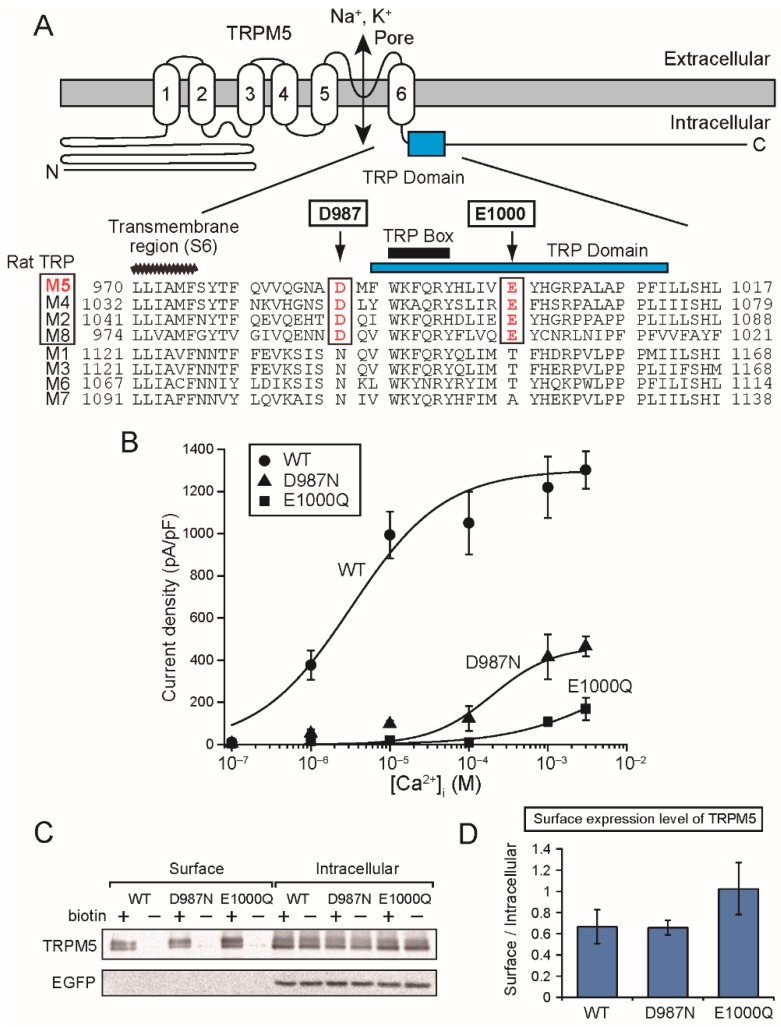
The mutations of the negatively-charged amino acid residues near and in the TRP domain reduced the Ca^2+^-sensitivity of rTRPM5. (**A**) Positions of the acidic amino acid residues (Asp^987^ (D987) and Glu^1000^ (E1000)) which were mutated. (Upper) The predicted membrane topology of rTRPM5 and the position of TRP domain in the C-terminal tail. (Lower) An alignment of amino acid sequences around the TRP domain of rat TRPM (rTRPM) channels. The aspartate and the glutamate of rTRPM5 are conserved in rTRPM4 (GenBank #NP_001129701.1), rTRPM2 (NP_001011559.1) and rTRPM8 (NP_599198.2) but not in rTRPM1 (NP_001032823.1), rTRPM3 (NP_001178491.1), rTRPM6 (XP_006223728.1) nor rTRPM7 (NP_446157.2). (**B**) CRCs for the effect of Ca^2+^ on the rTRPM5 current densities at +100 mV. EC_50_ for Ca^2+^ of WT rTRPM5 and D987N mutant were 3.25 and 196 µM, respectively. EC_50_ for Ca^2+^ of E1000Q was unable to be estimated but it seems to be at least more than 100 µM. (**C**) A result of a biotinylation assay in order to evaluate the surface expression level of rTRPM5. The proteins of rTRPM5 and EGFP were detected by Western blotting. The biotinylated rTRPM5 (biotin+, Surface) is indicative of rTRPM5 which was expressed in the plasma membrane. Expression levels of EGFP in the intracellular fractions indicate the transfection efficiencies, and no signal of EGFP in the surface fractions indicates that intracellular proteins were not biotinylated. In order to monitor nonspecific binding, lysates of cells which were not treated with biotin were also subjected to precipitations with the streptavidin-agarose beads (biotin−). (**D**) Signal ratios of the surface rTRPM5 to the intracellular rTRPM5 as an indication of the surface expression levels of rTRPM5. The expression levels of WT rTRPM5 and mutants did not differ significantly (*n* = 3).

Next, we examined the effects of mutations of the negatively-charged amino acid residues near and in the TRP domain on the Ca^2+^-sensitivity of rTRPM5 (Figure 5A). Corresponding amino acid residues of hTRPM4, rTRPM4 and rTRPM5, which were mutated in this study, are summarized in Table 1. Asp^987^ is located immediately before the TRP domain and was mutated to asparagine (D987N). Glu^1000^ is located in the TRP domain and was mutated to glutamine (E1000Q). As TRPM5 was inhibited by Zn^2+^ [17], Zn^2+^-sensitive currents were analyzed as rTRPM5 currents. Typical whole-cell currents are shown in Supplementary Figure S3B,C. The effects of different [Ca^2+^]_i_ was evaluated by using pipette solutions which contained different free Ca^2+^ concentrations. Similar to our previous report concerning rTRPM4 [6], the mutations of the aspartate and the glutamate (D987N, E1000Q) reduced the Ca^2+^-sensitivity of rTRPM5 (Figure 5B). The mutations did not reduce the surface expression of rTRPM5 (Figure 5C,D).

#### 2.2.2. The Ca^2+^-Sensitivity of rTRPM5 was Impaired by the Mutations of the Amino Acid Residues Which Were Proposed to form the Ca^2+^-Binding Site

**Figure 6:**
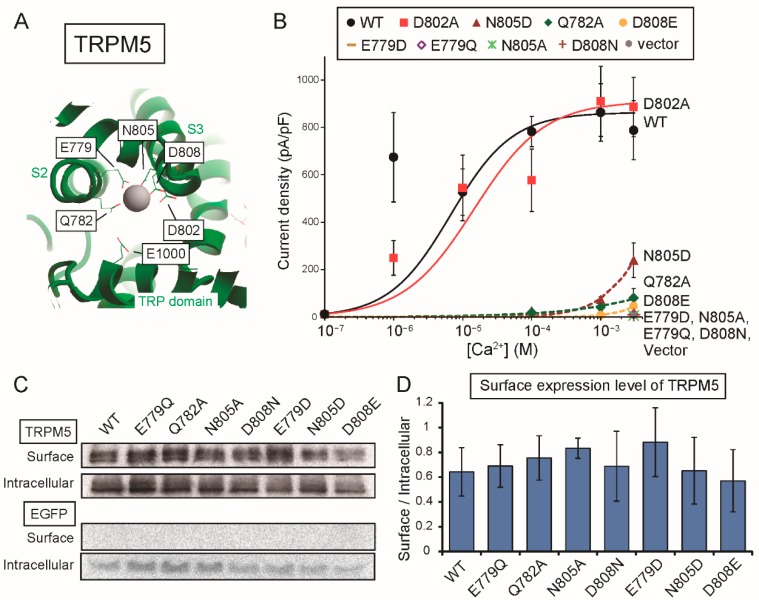
The amino acid residues of TRPM5 corresponding to those forming the Ca^2+^-binding site of TRPM4 were also necessary for the normal Ca^2+^-sensitivity of rTRPM5. (**A**) Numbers of corresponding amino acids of rTRPM5 are labeled in the illustration of the Ca^2+^-binding site of hTRPM4. (**B**) CRCs for the effect of Ca^2+^ on the current densities mediated by WT rTRPM5 (black circles), D802A (red squares), N805D (dark red triangles), Q782A (green diamonds), D808E (dark yellow circles), E779D (dark yellow horizontal bars), E779Q (purple open diamonds), N805A (light green asterisks), D808N (brown crosses) and the empty vector (gray circles) (*n* = 3 or 4 each). (**C**) A result of surface biotinylation assay. The proteins of rTRPM5 expressed in the plasma membrane were biotinylated and precipitated with streptavidin beads (Surface). Non-precipitated fractions contain intracellular proteins (Intracellular). (**D**) Signal ratios of the surface rTRPM5 to the intracellular rTRPM5 as an indication of the surface expression levels of rTRPM5. The mutants showed similar surface expression levels to WT rTRPM5 (*n* = 3).

We then examined the effects of mutations of the amino acid residues of the proposed Ca^2+^-binding site on the Ca^2+^-sensitivity of rTRPM5. As no three-dimensional structures of TRPM5 are available, corresponding amino acid residues of rTRPM5 are labeled on the structure of hTRPM4 [11] in Figure 6A. The four amino acid residues (Glu^779^, Gln^782^, Asn^805^, and Asp^808^) seem to contact with Ca^2+^, and the aspartate (Asp^802^) seems to not contact with Ca^2+^. They were mutated to alanine (Q782A, D802A and N805A), neutral amino acid residues (E779Q and D808N) or acidic amino acid residues (E779D, N805D and D808E). Firstly, only D802A mutation did not shift CRC so much in comparison with that of WT rTRPM5 (Figure 6B), although the mutation of the corresponding amino acid residue of rTRPM4 (D856A) shifted its CRC obviously (Figure 2B). All the other mutations severely impaired the sensitivity of rTRPM5, and many mutants (E779Q, E779D, N805A and D808N) were completely inactive even in the presence of 3 mM Ca^2+^_i_ (Figure 6B). We were unable to measure whole-cell currents in the presence of 10 mM Ca^2+^_i_ because the membrane of the cells became fragile under the condition. Biotinylation assays revealed that these mutations did not reduce surface expression levels of the mutant rTRPM5 (Figure 6C,D). These results indicate that the four amino acid residues, which were proposed to form the Ca^2+^-binding site of TRPM4, also form the Ca^2+^-binding site of rTRPM5. Additionally, it was also suggested that the aspartate at the entrance of the site (D802) does not play an important role in determining the Ca^2+^-sensitivity of rTRPM5 in comparison with the case of rTPRM4.

### 2.3. The Responsiveness to PI(4,5)P_2_ and PIP_3_ of rTRPM5 Differed from that of rTRPM4

**Figure 7:**
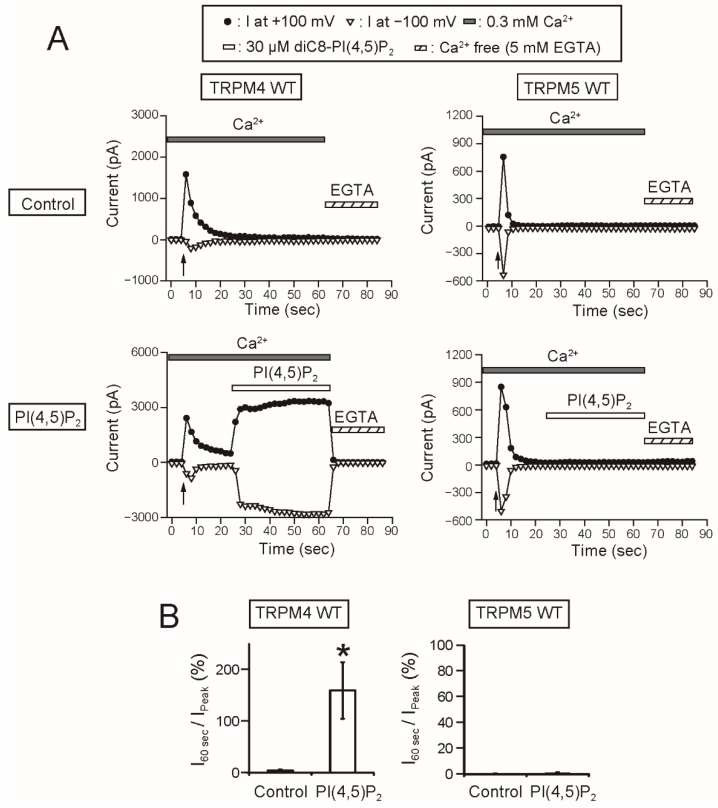
PI(4,5)P_2_ restored TRPM4 currents but not TRPM5 currents after their desensitization. (**A**) Typical time courses of the inside-out patch currents of WT rTRPM4 (left) or WT rTRPM5 (right) at +100 mV (filled circles) or −100 mV (open inverted triangles). The time of patch excisions were indicated by arrows. Twenty seconds after the patch excisions, 30 μM diC8-PI(4,5)P_2_ (water-soluble PI(4,5)P_2_) was applied (lower) or not applied (control, upper). The Ca^2+^ concentration of bath solutions (i.e., intracellular side, shaded bar) was 0.3 mM. At the end of measurements, the bath solution was changed to the Ca^2+^-free EGTA-containing solution (EGTA, hatched bar). (**B**) Ratios of the current amplitudes at 60 s after patch excision to the initial peak current amplitudes (I_60 sec_/I_peak_) at +100 mV in the absence (Control) or the presence (PI(4,5)P_2_) of 30 μM diC8-PI(4,5)P_2_ (*n* = 6–10). * *p* < 0.05 vs. Control. The current amplitudes of rTRPM4 in the presence of PI(4,5)P_2_ were 160% of the initial peak current amplitudes, but the current amplitudes of rTRPM5 in the presence of PI(4,5)P_2_ were negligible and similar to those in the absence of PI(4,5)P_2_.

Finally, we examined the effect of applications of phosphoinositides (PI(4,5)P_2_ and PIP_3_) on rTRPM5. Firstly, the applications of 30 μM PI(4,5)P_2_ to the cytosolic side of the inside-out patch membrane restored the rTRPM4 currents 100% or more (Figure 7). The 30 μM was reported to be a high enough concentration of PI(4,5)P_2_ to induce the maximal effect on TRPM4 [3,4,6]. However, the applications of the same concentration of PI(4,5)P_2_ under the same condition did not restore rTRPM5 currents after their desensitization at all (Figure 7).

**Figure 8:**
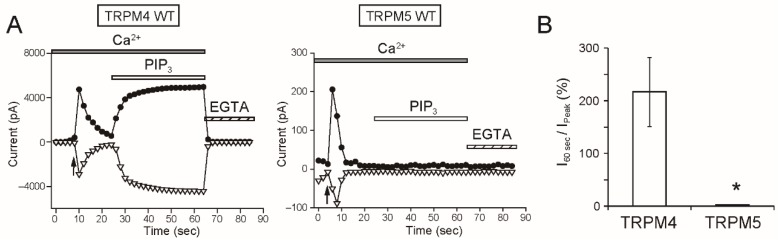
PIP_3_ also did not restore TRPM5 currents. (**A**) Typical time courses of the inside-out patch currents of WT rTRPM4 (left) or WT rTRPM5 (right) at +100 mV (filled circles) or −100 mV (open inverted triangles). The time of patch excisions were indicated by arrows. Twenty seconds after the patch excisions, 30 μM diC8-PI(3,4,5)P_3_ (water-soluble PIP_3_) was applied. The Ca^2+^ concentration of bath solutions (i.e., intracellular side, shaded bar) was 0.3 mM. (**B**) A summary of ratios of the current amplitudes of rTRPM4 and rTRPM5 in the presence of PIP_3_ at 60 s after patch excisions to their initial peak current amplitudes (I_60 sec_/I_peak_) at +100 mV. *n* = 4 and 6. * *p* < 0.05 vs. TRPM4.

PIP_3_ is another phosphoinositide in the plasma membrane, though the concentration of PIP_3_ is lower than that of PI(4,5)P_2_ [2]. PIP_3_ was also reported to bind to TRPM4 [16] and restore the Ca^2+^-sensitivity of TRPM4 [3]. The applications of PIP_3_ restored rTRPM4 currents also in this study (Figure 8). However, the applications of PIP_3_ also did not restore rTRPM5 currents (Figure 8).

## 3. Discussion

Firstly, the results in this study provide the functional evidence that the four amino acid residues (Glu^822^, Gln^825^, Asn^859^ and Asp^862^ of rTRPM4), which bound to Ca^2+^ in the cryo-EM structure of hTRPM4 [11], form the Ca^2+^-binding site of rTRPM4. Secondly, although any three-dimensional structures of TRPM5 are not available, the results in this study also indicate that the same amino acid residues (Glu^779^, Gln^782^, Asn^805^ and Asp^808^ of rTRPM5) form the Ca^2+^-binding site of rTRPM5. The Ca^2+^-binding sites of the other Ca^2+^-sensitive TRPM channels (TRPM2 and TRPM8) were also reported to be formed by the same four amino acid residues [13,14,15]. Therefore, the Ca^2+^-binding site was revealed to be conserved in all the Ca^2+^-sensitive TRPM channels as the information on the Ca^2+^-binding site of TRPM5 was added by this study.

There may be a difference between the Ca^2+^-binding site of rTRPM4 and that of rTRPM5 only in the involvement of the aspartate (Asp^856^ of rTRPM4 and Asp^802^ of rTRPM5) at the entrance of the Ca^2+^-binding site. The mutation of the aspartate to alanine reduced the Ca^2+^-sensitivity of rTRPM4 (D856A in Figure 2B), but the same mutation did not have an obvious effect on the Ca^2+^-sensitivity of rTRPM5 (D802A in Figure 6B). In the case of rTRPM4, Asp^856^ likely faces the pathway for Ca^2+^ as shown in the structure of hTRPM4 [11] and its negative charge may facilitate the access of Ca^2+^ to its binding site. On the other hand, in the case of rTRPM5, Asp^802^ might be oriented to a different direction or be covered by other amino acid residues, so that Asp^802^ might fail to facilitate the access of Ca^2+^. The reason why Asp^802^ plays a less important role in the Ca^2+^-sensitivity of rTRPM5 will be revealed when the three-dimensional structure of TRPM5 is unveiled.

An important novel finding in this study is that the negatively-charged amino acid residues near and in the TRP domain are also necessary for the normal Ca^2+^-sensitivity of rTRPM5. This finding raises the importance of these amino acid residues for understanding the mechanisms of TRPM channel opening by Ca^2+^. The glutamate in the TRP domain of the cryo-EM hTRPM4 structure was shown to be located in the pathway leading to the Ca^2+^-binding site from the cytoplasmic space [11]. Therefore, the negative charge of the glutamate is thought to increase the accessibility of Ca^2+^ to the site [11]. However, it should be noted that the corresponding glutamate of human TRPM2 (hTRPM2, Glu^1073^) was shown to participate in the coordination of Ca^2+^ together with the four amino acid residues in S2 and S3 according to the Ca^2+^-bound and “open” structure of hTRPM2 [14]. The structure of hTRPM4, which was referenced in this study, is a Ca^2+^-bound structure but is in a “closed” state [11]. Therefore, if the Ca^2+^-bound and open structures of TRPM4 and TRPM5 are unveiled, the glutamate in TRPM4 and TRPM5 might be revealed to directly participate in the formation of the Ca^2+^-binding site in addition to the four amino acid residues in S2 and S3.

The mutation of the aspartate (Asp^1049^ of rTRPM4 and Asp^987^ of rTRPM5) just before the TRP domain also reduced Ca^2+^-sensitivities of rTRPM4 [6] and rTRPM5 (Figure 5B). The aspartate is located at the beginning of the α-helix containing the TRP domain just after the bend below S6 (Figure 1A and Figure 5A). Therefore, the mutation of the aspartate might move the direction of the TRP domain, and the consequent displacement of the glutamate in the TRP domain might be a reason for the reduced Ca^2+^-sensitivities of the mutants.

As a remarkable finding, the intracellular applications of PI(4,5)P_2_ and PIP_3_ did not restore the rTRPM5 currents although they restored the rTRPM4 currents completely (Figure 7 and Figure 8). These experiments were conducted under the same conditions. We cannot exclude the possibility that PI(4,5)P_2_ and PIP_3_ can restore the TRPM5 currents under other experimental conditions. However, from the beginning, the effect of PI(4,5)P_2_ on TRPM5 was reported not to be so strong [5]. Therefore, when we take both observations from elsewhere [5] and our results into account, we consider it will be most likely that the regulation of the Ca^2+^-sensitivity of TRPM5 by PI(4,5)P_2_ is limited, if any, in comparison with the case of TRPM4.

The PI(4,5)P_2_-binding site of full-length TRPM4 has not been elucidated. Initially, putative pleckstrin homology (PH) domains in its cytosolic C-terminal tail were suggested to be candidates for the PI(4,5)P_2_-binding site [4]. However, the putative PH domains are less likely to be the PI(4,5)P_2_-binding site of TRPM4 because the domains appear not to be accessible based on the cryo-EM structure [9]. A more feasible candidate for the PI(4,5)P_2_-binding site of TRPM4 is pre-S1 region in the cytosolic N-terminal region, as PI(4,5)P_2_ and PIP_3_ were shown to bind to the pre-S1 region fragment of hTRPM4 by surface plasmon resonance measurements [16]. Arg^755^ and Arg^767^ of hTRPM4 were shown to be crucial in the interaction with PI(4,5)P_2_ and PIP_3_. The first arginine is conserved in rTRPM4. However, the second arginine is not conserved in rTRPM4. There is no amino acid residue in the position of the second arginine in rTRPM4 based on an alignment of amino acid sequences of hTRPM4 and rTRPM4. Therefore, it is not certain whether PI(4,5)P_2_ binds to the pre-S1 region of rTRPM4. However, the PI(4,5)P_2_ binding site of TRPM8 has been reported to be formed by the combination of its pre-S1 region and other regions [15]. Similarly, in the case of full-length TRPM4, the role of the second arginine, shown in the fragment of hTRPM4, may be played by another basic amino acid residue in another region. Although the pre-S1 region of TRPM4 may be involved in the binding of PI(4,5)P_2_, it should be noted that the similarity of the pre-S1 region between TRPM channels is quite low. It has already been pointed out elsewhere that the PI(4,5)P_2_ binding site of TRPM8 is not conserved in other TRPM channels [15]. In the pre-S1 region of TRPM5, the basic amino acid residues which were suggested to be necessary for the interaction with PI(4,5)P_2_ in TRPM4 and TRPM8 are not conserved. That might be a reason for the negligible effect of PI(4,5)P_2_ on rTRPM5. Conversely, based on the finding that the effect of PI(4,5)P_2_ on rTRPM5 was negligible, the comparisons of amino acid sequences or structures between TRPM4 and TRPM5 may lead to revealing of the PI(4,5)P_2_ binding site of TRPM4.

In this study, we did not examine the relationship between the activation of TRPM4 by Ca^2+^ and the regulation by PI(4,5)P_2_. In this study and elsewhere [11], the amino acid residues forming the Ca^2+^-binding site of TRPM4 have been revealed. Therefore, by analyzing PI(4,5)P_2_-affinities of their mutants and also the effects of PI(4,5)P_2_ on the maximum currents of the mutants, the molecular understanding of the relationship between the activation by Ca^2+^ and the regulation by PI(4,5)P_2_ will be expanded.

It is difficult to state clearly the physiological meaning of the difference in the effect of PI(4,5)P_2_ on TRPM4 and TRPM5. That is because even the physiological meaning of the interaction with PI(4,5)P_2_ in TRPM4 in native cells has not yet been clearly explained as far as we know, although it is certain that PI(4,5)P_2_ is necessary for the high activity of TRPM4. However, PI(4,5)P_2_-insensitivity of TRPM5 might be advantageous for its function in type II taste cells where TRPM5 is natively expressed. In the taste cells, TRPM5 is activated by Ca^2+^_i_, which is released from the endoplasmic reticulum by inositol trisphosphate (IP_3_) signaling, and the opening of TRPM5 depolarizes the membrane potential, resulting in release of transmitters [18]. The IP_3_ is produced by hydrolysis of PI(4,5)P_2_, which is mediated by phospholipase C (PLC), when tastants bind to G-protein coupled taste receptors. Therefore, due to the low dependency of TRPM5 on PI(4,5)P_2_, the Ca^2+^-sensitivity of TRPM5 might not be affected by the reduction of PI(4,5)P_2_ concentration in the plasma membrane, which might occur due to the PLC-mediated hydrolysis of PI(4,5)P_2_. However, in order to prove this theory, it needs to be demonstrated that the PI(4,5)P_2_ concentration in the plasma membrane around TRPM5 is actually reduced after the stimulation by tastants.

In conclusion, this study revealed the following three major findings regarding the functional mechanisms of TRPM4 and TRPM5: (1) that both Ca^2+^-binding sites of TRPM4 and TRPM5 are formed by the same four amino acid residues in S2 and S3; (2) the glutamate in the TRP domain is also necessary for their normal Ca^2+^-sensitivities; (3) and finally, the regulation of their Ca^2+^-sensitivities by PI(4,5)P_2_ may be restricted to TRPM4. These findings based on the functional analyses will be beneficial for the further understanding of the structure-function relationships of TRPM4 and TRPM5 even after the three-dimensional structure of TRPM5 is unveiled.

## 4. Materials and Methods

### 4.1. Animal Ethics Approval

Animal experiments were performed in accordance with guidelines and protocols approved by the Institutional Animal Care and Use Committee, Hokkaido University (The project identification code is #13-0212, approved on 27 January 2014).

### 4.2. Molecular Cloning and Site-Directed Mutagenesis

A male BN/SsNSlc rat (five weeks old) was euthanized by CO_2_ inhalation. RNA was extracted from the tongue epithelia containing circumvallate papillae and foliate papillae using NucleoSpin RNA II (Takara Bio, Otsu, Japan). Complementary DNA (cDNA) was synthesized using PrimeScript II Reverse Transcriptase (Takara Bio) and an oligo dT primer. The full-length of the open reading frame of rat *Trpm5* cDNA was amplified by PCR using a high fidelity polymerase (PrimeSTAR GXL, Takara Bio) and the following primers: 5’-GCA AGG GAG GAA CAC AGC CTG AAG TAG-3’ (Forward primer in 5’ UTR) and 5’-GAC GTA AGT AGC CCC ATC CAG GCA G-3’ (Reverse primer in 3’ UTR). These primers were designed based on the predicted *rTrpm5* mRNA sequences (Genbank #XM_017589501.1 and XM_008760160.2) which were derived from a genomic sequence. The PCR product, amplified *rTrpm5* cDNA, was cloned in pGEM T-Easy vector (Promega, Fitchburg, WI, USA) and sequenced. The *rTrpm5* cDNA (Genbank #LC469323) which contained no PCR errors was subcloned into a bicistronic expression vector, pIRES2-EGFP (Takara Bio), from which both EGFP and a protein encoded by the inserted gene can be expressed. The pIRES2-EGFP vectors containing *rTrpm4* cDNA had been made in the previous study [6]. Site-directed mutagenesis of *rTrpm5* and *rTrpm4* cDNA in pIRES2-EGFP was accomplished using the PrimeSTAR Mutagenesis Basal kit (Takara Bio). The mutations were verified by sequencing.

### 4.3. Cell Culture and Transfection

HEK 293T cells were obtained from the RIKEN BioResource Research Center through the National Bio-Resource Project of the MEXT, Tokyo, Japan. HEK 293T cells were cultured in DMEM (Dulbecco’s modified Eagle’s medium; Sigma-aldrich, St. Louis, MO, USA) supplemented with 10% FBS (Thermo Fisher Scientific, Waltham, MA, USA) and penicillin/streptomycin (100 U/mL and 100 μg/mL, respectively, Thermo Fisher Scientific) at 37 °C in a 5% CO_2_ incubator. Cells were transiently transfected with plasmids using TransIT-293 Transfection Reagent (Takara Bio). Two days after the transfection, the cells were used for Western blot analyses and biotinylation assays. For patch clamp experiments, the cells were plated on coverslips the following day of the transfection. After at least 3 h of further culturing, whole-cell patch-clamp recordings were made from EGFP-positive cells. Inside-out patch recordings were performed on the following day of the cell plating on coverslips.

### 4.4. Electrophysiology

Patch-clamp recordings were performed as described previously [6,19]. Briefly, the conditions for inside-out current recordings are as follows. The pipette solution for the inside-out recordings was composed of 145 mM NaCl, 1 mM CaCl_2_, 1 mM MgCl_2_ and 10 mM HEPES (2-[4-(2-hydroxyethyl)piperazin-1-yl]ethanesulfonic acid) (pH = 7.4, with NaOH). Bath solutions contained 145 mM NaCl and appropriate concentrations of CaCl_2_ or 5 mM EGTA (Ethylene glycol-bis(2-aminoethylether)-*N*,*N*,*N*′,*N*′-tetraacetic acid) for a Ca^2+^-free solution. Macroscopic currents under the inside-out configuration were recorded using the ramp pulses. The holding potential was −60 mV (the intracellular side is negative), and the ramp pulses from −100 to +100 mV with durations of 400 ms were applied every 2 s. The currents were filtered at 1 kHz and sampled at 5 kHz. Water soluble phosphoinositides, diC8-PI(4,5)P_2_ and diC8-PI(3,4,5)P_3_, were obtained from Cellsignals (Columbus, OH, USA) and dissolved in the bath solution.

The whole-cell patch clamp recordings for rTRPM5 current measurements were performed under the conditions described below. The pipette solution was composed of 130 mM CsCl, 10 mM BAPTA (1,2-bis(o-aminophenoxy)ethane-*N*,*N*,*N*’,*N*’-tetraacetic acid), 4 mM ATP disodium salt, 10 mM HEPES (pH = 7.3, adjusted with CsOH) and appropriate amounts of MgCl_2_ and CaCl_2_ in order to make solutions which contained 10^−3^ M free Mg^2+^ and 0–10^−4^ M free Ca^2+^. The free Mg^2+^ and Ca^2+^ concentrations were calculated using the CaBuf software (Guy Droogmans, KU Leuven, Leuven, Belgium). The pipette solutions containing 1 and 3 mM CaCl_2_ were made without BAPTA. The extracellular solution was composed of 145 mM NaCl, 2 mM MgCl_2_ and 10 mM HEPES (pH = 7.4, with NaOH). In most experiments, the holding potential was −60 mV and the ramp pulses from −100 to +100 mV with durations of 400 ms were applied every 2 s. The currents were filtered at 1 kHz and sampled at 5 kHz. One minute after the start of the whole-cell recording, 50 μM ZnCl_2_ was applied through the bath solution. Current densities were calculated by dividing the current amplitudes by the cell capacitances (pA/pF). As an indication of rTRPM5 current densities, the Zn^2+^-sensitive current densities were calculated by subtracting the current densities in the presence of Zn^2+^ from the peak current densities. All experiments were conducted at room temperature.

### 4.5. Biotinylation Assay and Western Blotting

The transfected HEK293T cells were washed twice with ice cold D-PBS-CM (Dulbecco’s phosphate buffered saline with 1 mM MgCl_2_ and 0.1 mM CaCl_2_) and incubated 15 min with 1.0 mg/ml EZ-link sulfo-NHS-s-s-biotin (Thermo Fisher Scientific) in cold biotinylation buffer (10 mM triethanolamine, 2 mM CaCl_2_, 150 mM NaCl, pH 9.0, adjusted with HCl) with gentle agitation at 4 °C. The cells were washed twice with a quenching buffer (100 mM glycine in D-PBS-CM), then rinsed once with D-PBS, scraped in cold D-PBS and pelleted at 290×*g* for 1 min at 4 °C. The cells were lysed in a lysis buffer (150 mM NaCl, 50 mM HEPES, 1.0 mM EGTA, 1.5 mM MgCl_2_, pH = 7.4, 1.0% Triton X-100, 10% glycerol and a protease inhibitor cocktail (P8340, Sigma-Aldrich)). The lysates were sonicated using Bioruptor (BM Equipment, Tokyo, Japan) or Ultrasonic homogenizer (UH50, SMT, Tokyo Japan). The lysates were centrifuged for 10 min at 14,000× *g* at 4 °C. The protein concentrations of the lysates were measured by using DC Protein Assay (Bio-Rad, Hercules, CA, USA) and adjusted to 100 μg in 150 μL with the lysis buffer. Streptavidin-agarose beads (Sigma-Aldrich) were added to the protein extracts. The mixtures were rotated at 20 rpm for 2 h at 4 °C. The beads were pelleted by brief centrifugation (650× *g* for 1 min). The supernatants were taken as the unbound intracellular fractions, mixed with one fourth volume of 4× Laemmli sample buffer containing 10% β-mercaptoethanol, and heated at 50 °C for 15 min. The beads were washed three times with D-PBS containing 0.5% Triton X-100. The biotinylated proteins were eluted from the beads by mixing the beads with 4× Laemmli sample buffer containing 10% β-mercaptoethanol and heating the mixtures at 50 °C for 15 min (Surface fractions).

The intracellular fractions (10 μg protein = 10% of total protein used for one sample) and the surface fractions were separated by SDS-PAGE and transferred to a PVDF membrane. After incubation in blocking buffer containing 3% skim milk, the blots were treated with the diluted anti-TRPM5 rabbit antibody (1:2000, #ACC-045, Alomone labs, Jerusalem, Israel) or anti-GFP rabbit antibody (1:500, #598, MBL, Nagoya, Japan) and then with horseradish peroxidase-conjugated anti-rabbit IgG antibody (1:1000, GE Healthcare, Buckinghamshire, UK) as the secondary antibody. The chemiluminescent signals were produced by using Immobilon Forte (Merk, Burlington, MA, USA) and detected by a single-lens reflex camera (EOS kiss x7, Canon, Tokyo, Japan).

### 4.6. Data Analysis

Concentration-response curves were obtained by fitting the averages of current amplitudes or current densities with the Hill equation:

$$I=I_{max}\times \frac{C^{n}}{EC_{50}{}^{n}+C^{n}},$$

where *I_max_* is the maximal current amplitude or the maximal current density, *C* is the concentration of Ca^2+^, *EC*_50_ is the half maximal effective concentration and *n* is the Hill coefficient.

All data are expressed as means ± S.E. The statistical analyses were performed using Student’s *t* test, Welch’s *t* test or Dunnett’s test as appropriate. A value of *p* < 0.05 was considered significant.

## Figures and Tables

**Table 1:** Corresponding amino acid residues.

Human TRPM4	Rat TRPM4	Rat TRPM5
Glu (E) 828	E822	E779
Gln (Q) 831	Q825	Q782
Asp (D) 862	D856	D802
Asn (N) 865	N859	N805
Asp (D) 868	D862	D808
Asp (D) 1055	D1049	D987
Glu (E) 1068	E1062	E1000

## Supplementary Materials

The following are available online at https://www.mdpi.com/1422-0067/20/8/2012/s1.

## Abbreviations

BAPTA	1,2-bis(o-aminophenoxy)ethane-*N*,*N*,*N*’,*N*’-tetraacetic acid
Ca^2+^_i_	Intracellular Ca^2+^
[Ca^2+^]_i_	Intracellular Ca^2+^ concentration
cDNA	Complementary DNA
CRC	Concentration-response curve
Cryo-EM	Cryo-electron microscopy
DMEM	Dulbecco’s modified Eagle’s medium
D-PBS	Dulbecco’s phosphate buffered saline
EC_50_	Half maximal effective concentration
EGFP	Enhanced green fluorescent protein
EGTA	Ethylene glycol-bis(2-aminoethylether)-*N*,*N*,*N*′,*N*′-tetraacetic acid
HEPES	2-[4-(2-hydroxyethyl)piperazin-1-yl]ethanesulfonic acid
IP_3_	Inositol trisphosphate
PI(4,5)P_2_	Phosphatidylinositol 4,5-bisphosphate
PLC	Phospholipase C
Sx	Transmembrane segment x
TRPM	Transient receptor potential melastatin
UTR	Untranslated region
