# Supplementary material for: TRPM4 and TRPM5 Channels Share Crucial Amino Acid Residues for Ca2+ Sensitivity but Not Significance of PI(4,5)P2
Source: Int J Mol Sci. 2019 Apr 24;20(8):2012. doi: 10.3390/ijms20082012 (PMC6514954; doi:10.3390/ijms20082012)
Supplement: Supplementary file 1 [file ijms-20-02012-s001.pdf]

International Journal of
Molecular Sciences

MDPI

Supplementary Materials for "TRPM4 and TRPM5 channels share crucial amino acid residues for Ca^{2+} sensitivity but not significance of $PI(4,5)P_2$", Yamaguchi et. al.

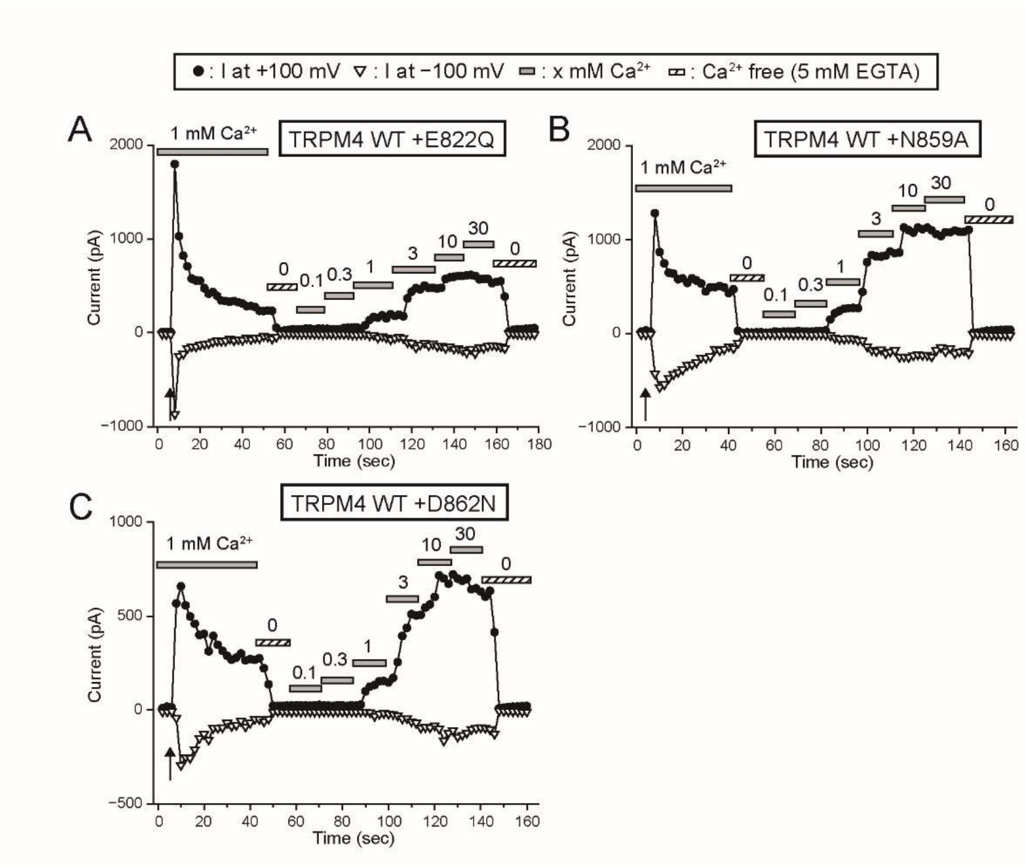

Figure 1. Typical time courses of the changes in the currents at +100 mV (filled circles) and −100 mV (open inverted triangles) measured from the cells which were cotransfected with WT and E822Q (**A**), WT and N859A (**B**), and WT and D862N (**C**).

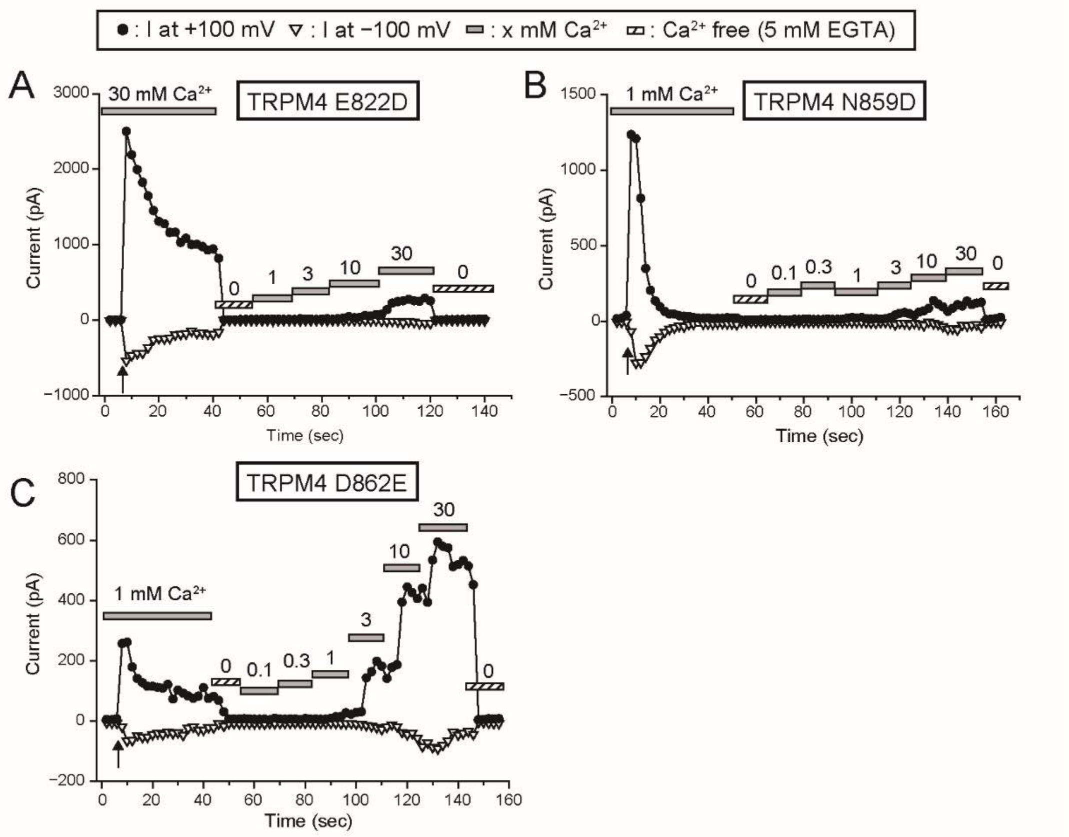

Figure S2. Typical time courses of the changes in the currents at +100 mV (filled circles) and −100 mV (open inverted triangles) mediated by rTRPM4 E822D (**A**), N859D (**B**), and D862E (**C**).

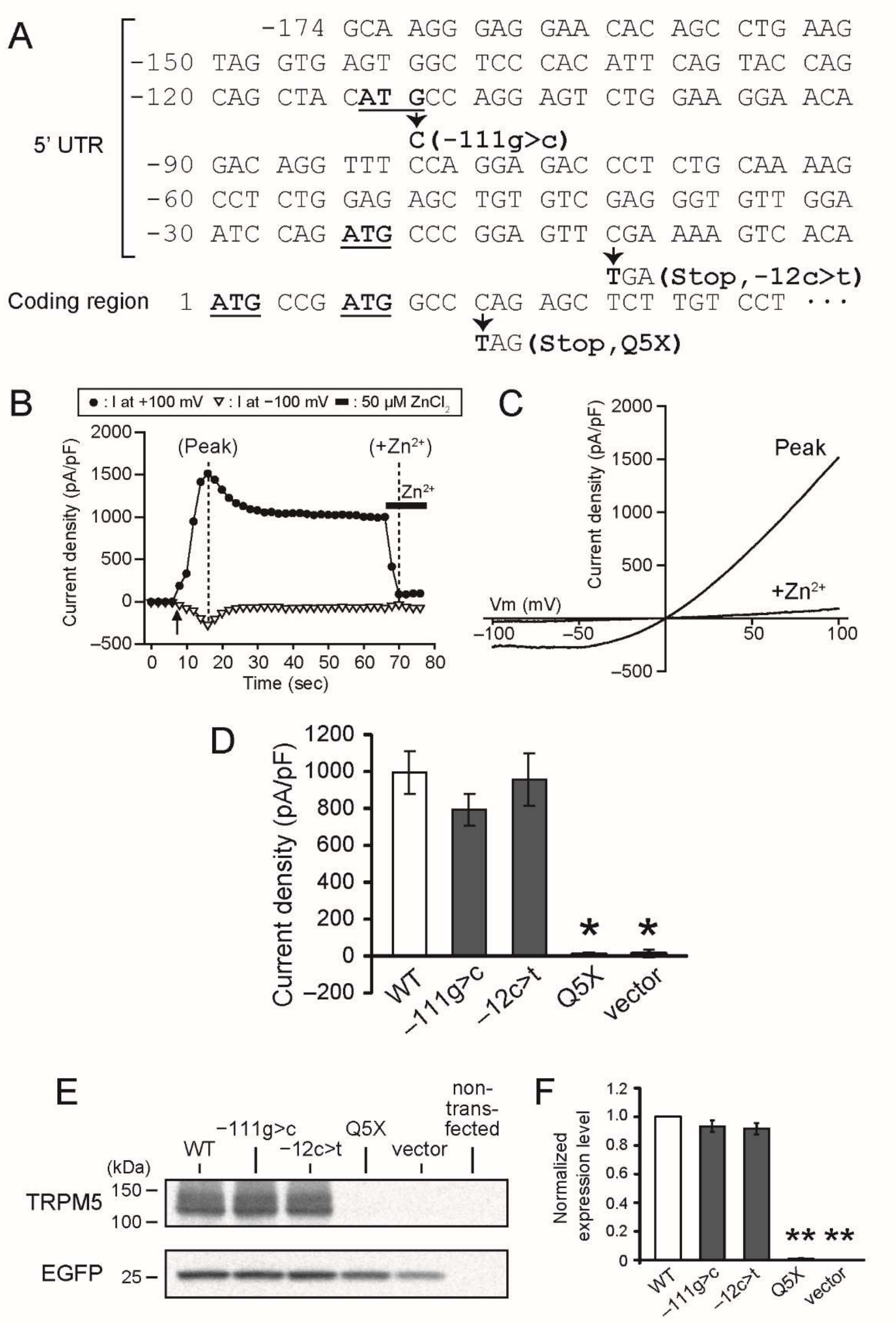

Figure S3. The translation of rTRPM5 does not start, at least mainly, from the possible initiation codons in 5′ untranslated region (UTR). (**A**) The nucleotide sequence of the cloned rat *Trpm5* complimentary DNA (cDNA) (Genbank #LC469323). The nucleotides in 5′ UTR are numbered in a retrograde fashion, and 1 is given to the adenine of ATG, which is registered to be the initiation codon in the rat *Trpm5* cDNA (Genbank #NM_001191896.1). In order to examine whether translation starts from the possible initiation codons (ATG) in 5′ UTR, the guanine of the first ATG was mutated to cytosine (−111g>c) or an in-frame stop codon was added after 12 nucleotides by mutating the cytosine to thymine (−12c>t). In order to examine whether the translation starts from the supposed initiation codon (ATG from 1 to 3) or the next ATG (from 7 to 9), the codon for Gln^5 was replaced by a stop codon (Q5X). (**B**) Typical time courses of WT rTRPM5 whole cell current

densities at +100 mV (filled circles) and −100 mV (inverted open triangles). The pipette solution contained 10^{-5} M free Ca^{2+}. The arrow indicates the time of establishment of whole-cell patch configuration. One minute after the start of the whole-cell recording, 50 μM $ZnCl_2$ was applied through the bath solution. The data points used for Figure S3C were indicated by the dashed lines. (**C**) Current-voltage relationship of the peak currents and the currents after Zn^{2+} was applied. (**D**) A summary for the Zn^{2+}-sensitive currents densities measured from the cells which were transfected with WT *rTrpm5*, −111g>c mutant, −12c>t mutant, Q5X mutant, and empty vector (n = 5–15). Statistical significance was analyzed by Dunnett's test (vs. WT, * $p < 0.05$). Zn^{2+}-sensitive currents densities were calculated by subtracting the remaining current densities in the presence of Zn^{2+} from the peak current densities. The current densities measured from the cells which were transfected with −111g>c mutant and −12c>t mutant were not significantly different from that measured from the cells which were transfected with WT *rTrpm5*. That indicates that the expression of functional rTRPM5 was not affected by eliminating the influence of the two ATG in 5′UTR. (**E**) A result of western blot analysis. The expressions of enhanced green fluorescent protein (EGFP) indicate the transfection efficiencies. The protein of rTRPM5 was not detected only from the cells which were transfected with Q5X mutant. (**F**) A summary of rTRPM5 expression levels which were normalized to that of WT. Statistical significance was analyzed by Dunnett's test (vs. WT, ** $p < 0.01$). The rTRPM5 expression levels in the cells which were transfected with −111g>c mutant and −12c>t mutant were not significantly different from that in the cells which were transfected with WT *rTrpm5*. That indicates that the expression of rTRPM5 was not affected by eliminating the influence of the two ATG in 5′UTR. The rTRPM5 expression level in the cells which were transfected with Q5X mutant was similar to that in the cells which were transfected with the empty vector (vector). In summary, these results indicate that the translation of rTRPM5 starts from the supposed initiation codon (ATG from 1 to 3) or the next ATG (from 7 to 9).
